# Targeting CD148 for Antithrombotic Therapy: Functional and Molecular Evaluation of AKB‐9778

**DOI:** 10.1002/prp2.70301

**Published:** 2026-07-31

**Authors:** Lina El Badaoui, Stipo Jurcevic, Yotis A. Senis, Alastair J. Barr

**Affiliations:** ^1^ School of Life Sciences University of Westminster London UK; ^2^ Centre for Cardiovascular and Nutrition Research, INSERM 1263, INRAE 1260, Faculty of Medicine Aix‐Marseille University Marseille France

**Keywords:** AKB‐9778, CD148, molecular docking, platelets, PTPRJ, razuprotafib, receptor‐type protein tyrosine phosphatase, thrombosis, T‐TAS, VE‐PTP

## Abstract

CD148 is the primary receptor‐type protein tyrosine phosphatase (PTP) regulating platelet activation, and its inhibition has been proposed as a novel anti‐thrombotic strategy with potentially lower bleeding risk than current therapies. However, selective and potent inhibitors of CD148 are currently lacking. AKB‐9778 (razuprotafib), a potent inhibitor of the closely related vascular endothelial‐PTP (VE‐PTP), has also been reported to inhibit CD148. This study investigated the effect of AKB‐9778 on in vitro whole‐blood thrombogenicity, examined the molecular basis of its interaction with the CD148 catalytic domain using molecular docking, and assessed inhibitor selectivity with an in vitro phosphatase assay. In a total‐thrombus formation analysis system (T‐TAS), AKB‐9778 partially inhibited thrombus formation, reducing AUC_10_ by 28%, without significantly affecting occlusion start time or occlusion time. The inhibitor did not alter collagen‐stimulated CD62P platelet surface expression or PAC‐1 binding to the activated integrin αIIbβ3. Molecular docking using CB‐Dock predicted a binding configuration in which the phenylsulfamic acid group of AKB‐9778 is orientated towards the catalytic cysteine, with additional conformations reflecting ligand flexibility. Site‐directed mutagenesis of Tyr1071, Gln1283, His1206, and Asn1073 to alanine reduced the inhibitory effect in phosphatase assays, supporting interaction with these residues. Notably, the IC_50_ of AKB‐9778 against CD148 was approximately 300‐fold higher than previously reported values, consistent with the wide variability across studies. Despite this reduced potency, these findings support the concept of CD148 inhibition as a potential anti‐thrombotic strategy and suggest that AKB‐9778 may serve as a useful prototype for developing more selective and efficacious CD148 inhibitors.

**Abbreviations**
BAPA: benzylsulfonyl‐d‐Arg‐Pro‐4‐amidino benzylamide
CRP: collagen‐related peptide cross‐linked
PAR‐1: protease‐activated receptor
PTP: protein tyrosine phosphatase
SAP‐1: stomach cancer‐associated protein tyrosine phosphatase‐1
T‐TAS: total‐thrombus formation analysis system
VE‐PTP: vascular endothelial protein tyrosine phosphatase

## 1 Introduction

CD148 (density‐enhanced phosphatase‐1), encoded by the *receptor‐type protein‐tyrosine phosphatase J* gene (*PTPRJ*), is the most abundant receptor‐type PTP expressed on platelets and plays a central role in regulating platelet activation and thrombus formation [1]. Knockout of CD148 in mice results in impaired thrombosis, but normal hemostasis. In conventional CD148 knockout mice, in vivo thrombosis assays demonstrated that thrombus formation was delayed, peak thrombus size was significantly reduced, and thrombi receded more rapidly [1]. Further studies analyzing megakaryocyte lineage‐specific CD148 knockout mice using in vivo and ex vivo models of thrombosis and hemostasis revealed that thrombus formation was compromised while normal hemostasis was observed [2]. Use of the microfluidic total thrombus‐formation analysis system (T‐TAS) in the study indicated that coagulation is unaltered and that the defect in thrombus formation is intrinsic to the platelets. Based on these findings, inhibition of CD148 has been suggested as a potential anti‐thrombotic strategy, which may reduce the significant bleeding risk associated with existing drugs [2, 3, 4].

Evidence from human genetic studies supports this concept. Marconi and colleagues reported that patients with a bi‐allelic loss of the *PTPRJ* gene, which results in almost complete loss of CD148 at the protein level, are affected by spontaneous bleeding and impaired platelet responses to agonists of the GPVI collagen receptor [5]. While a study by Rollin and colleagues [6], of the missense loss‐of‐function SNPs Q276P (rs1566734) and R326Q (rs1503185), found that platelets from individuals heterozygous for the minor alleles had reduced responsiveness to collagen in platelet aggregation tests. Similar results were obtained with antibodies activating platelets via the FcγRIIA receptor. The SNPs, which are in the ectodomain of CD148, are not associated with a change in CD148 expression [6, 7]. These findings are consistent with a model in which reduced CD148 activity leads to diminished activation of Src‐family kinases and impaired platelet signaling. However, these polymorphisms have not been clearly associated with protection from thrombotic events, as existing studies have been underpowered to detect such effects.

The challenges of targeting PTPs—achieving cell permeability and selectivity—have been well documented [8, 9, 10]. A consequence of the highly positively charged nature of the phosphatase active site is that many inhibitors are charged anionic phosphate mimetics that have limited membrane permeability. In addition, the active site of PTPs is highly conserved across the family, thus inhibitors often lack selectivity and inhibit closely related family members. CD148 is a member of the R3 subgroup of receptor‐type PTPs [11, 12], which includes vascular endothelial‐PTP (VE‐PTP, PTPRB) and closely related enzymes PTPRO (GLEPP1), stomach cancer–associated protein tyrosine phosphatase–1 (SAP‐1, PTPRH), and PTPRQ. A highly potent sulfamic acid‐based VE‐PTP inhibitor, AKB‐9778 (razuprotafib), with picomolar potency and significant selectivity within the family has been reported. Due to the structural similarity between VE‐PTP and CD148, AKB‐9778 has also been reported to inhibit CD148 [13]; however, the functional consequences of CD148 inhibition by AKB‐9778 and the molecular basis of its interaction with the CD148 catalytic domain remain incompletely understood. Phase 2 clinical trials have been conducted with AKB‐9778 to investigate efficacy in treatment of diabetic macular oedema, ocular hypertension and symptoms associated with COVID‐19 infection [14, 15, 16, 17]. The compound, however, failed to progress beyond Phase 2. AKB‐9778 has also been investigated in a mouse model of obesity, where it was found to ameliorate obesity‐induced systemic inflammation by inhibiting PTPRO [18]. In a study investigating VE‐PTP inhibition on breast cancer vasculature and metastatic progression, AKB‐9778 slowed the growth of micrometastasis by limiting extravasation of tumor cells [19].

In the present study, we investigated the effect of AKB‐9778 on in vitro thrombus formation using T‐TAS, a microchip‐based whole blood flow chamber system that recapitulates key aspects of physiological thrombosis. T‐TAS has been extensively used for quantitating anti‐thrombotic effects of various agents [20]. We hypothesized that AKB‐9778 would inhibit thrombus formation through inhibition of CD148. To further characterize the mechanism of inhibition, we assessed platelet activation responses by flow cytometry and examined the molecular basis of inhibitor binding using cavity‐detection guided molecular docking (CB‐Dock) [21] and site‐directed mutagenesis combined with in vitro phosphatase assays.

## 2 Materials and Methods

### 2.1 Blood Samples

Venous blood was obtained from healthy volunteers (21–67 years) by forearm venepuncture at the University of Westminster. The volunteers had not taken any medication in the preceding two weeks that might have affected platelet function. All donors had normal platelet counts ranging from 196 to 272 × 10^9^/L. Samples were collected in citrated tubes for flow cytometry experiments and T‐TAS analysis of AR‐chips while. Collection tubes with the thrombin inhibitor, benzylsulfonyl‐d‐Arg‐Pro‐4‐amidino benzylamide (BAPA), were used for T‐TAS analysis of PL chips. Protocols were approved by the Ethical Review Board of the University of Westminster (ETH2122‐0605).

### 2.2 Antibodies

BD Pharmingen FITC Mouse Anti‐Human CD62P (Cat# 555523) (20 μL/test), BD FITC Mouse Anti‐Human PAC‐1 (Cat# 340507) (20 μL/test), FITC Mouse Anti‐Human CD41a clone: HIP8 Mouse BALB/c IgG1,κ (Cat# 555466) (5 μL/test), PE Mouse Anti‐Human CD62P Mouse BALB/c IgG1,k (555524) (20 μL/test), PE Mouse Anti‐Human CD41a, clone HIP8 (Cat# 555467) (20 μL/test), APC Mouse Anti‐Human CD61 clone VI‐PL2 (Ca# 564174) (5 μL/test), and Human Integrin alpha 2b/CD41 Alexa Fluor 647‐conjugated Mouse IgG1 (Cat# FAB7616R) (5 μL/test) were obtained from BD Biosciences (Wokingham, UK). Human DEP‐1/CD148 PE‐conjugated Antibody clone 143‐41 Mouse IgG1 (Cat# FAB1934P) (10 μL/test) from R&D systems (Abingdon, UK).

### 2.3 Chemicals

AKB‐9778 was obtained from either MedKoo Biosciences (Durham, NC, USA) or Chemgood (Henrico, VA, USA) and was dissolved in 100% DMSO to yield a 50 mM stock solution. Collagen‐related peptide cross‐linked (CRP; also referred to as CRP‐XL) was synthesized by Peptide Protein Research Ltd. (Fareham, UK) [22]. PAR1 activating peptide, aspirin, DiFMUP and sodium ortho vanadate were purchased from Sigma‐Aldrich (Poole, UK).

### 2.4 In Vitro Thrombus Formation

In vitro thrombus formation under flow conditions was analyzed with the total thrombus‐formation analysis system (T‐TAS) (Fujimori Kogyo Co Ltd., Shinjuku, Japan) using freshly taken whole blood, as described previously [23]. To assess primary hemostasis, blood collected in a BAPA tube was either untreated or pre‐treated with drugs or vehicle (0.01%) as indicated for 20 min at 37°C before being applied to the reservoir and flowed through collagen‐coated capillaries of the PL chip at a shear rate of 1500 s^−1^. To assess primary and secondary hemostasis, blood was collected in a citrate tube and flowed through the AR chip (coated with collagen and thromboplastin) at a shear rate of 600 s^−1^. The process of thrombus formation was monitored by flow pressure changes in the capillary. As thrombus formation proceeds the capillary is gradually occluded, increasing the flow pressure. The parameters: occlusion start time (OST), time to reach base pressure + 10 kPa, occlusion time (OT), time to reach 60 kPa, and area under the flow pressure curve (AUC_10_) over a 10‐min period were calculated automatically. If the pressure waveform reaches the occlusion pressure within 10 min, the area below the response curve up to the point of arrival is added to the area for the remaining time with the upper limit as the occlusion pressure, and the combined area is calculated as AUC. Measurements were done in duplicate.

### 2.5 Sysmex Blood Count

Complete blood count tests were performed using a Sysmex XP‐300 automated hematology analyzer.

### 2.6 Flow Cytometry

Whole blood (5 μL) was incubated with antibodies, AKB‐9778 or DMSO control for 20 min in PBS (calcium and magnesium‐free) prior to addition of agonist at the indicated and incubated for 20 min in the dark at room temperature. Cells were fixed with 300 μL of 1% paraformaldehyde (BD Cytofix) before analysis on an LSRFortessa X‐20 (BD Biosciences) flow cytometer. Results were analyzed using Diva software and FlowJo.

### 2.7 In Vitro Phosphatase Assays

Protein tyrosine phosphatase activity was monitored using the substrate 6,8‐difluoro‐4‐methylumbelliferyl phosphate (DiFMUP) whose dephosphorylated reaction product, DiFMU, is a strong fluorophore. Assays (80 μL) were run in black 96‐well plates in continuous (kinetic) mode using a buffer consisting of 50 mM Tris–HCl, pH 7.4, 100 mM NaCl, 2 mM DTT and 1 mM EDTA on a Fluostar Optima plate reader (BMG Labtech) with Ex/Em filters of 350‐10/450‐10. Proteins for PTP catalytic domains from CD148 (PTPRJ), VE‐PTP (PTPRB), PTPRO and PTP1B were purified as described previously [24]. Initial experiments determined suitable enzyme concentrations to achieve a linear reaction rate over 30 min. A substrate concentration corresponding to the *K*_*m*_ was used. The AKB‐9778 inhibitor was used at a range of concentrations and pre‐incubated for 10 min prior to starting the assay with the addition of DiFMUP. Sodium ortho‐vanadate (Na_3_VO_4_) was used as a positive control. Curve fitting for calculation of IC_50_ and Km values was performed with GraphPad Prism 10 (GraphPad Software Inc., San Diego, USA).

### 2.8 Molecular Docking

Molecular docking of AKB‐9778 to PTP catalytic domain structure was performed using the CB‐Dock web server (http://cao.labshare.cn/cb‐dock/, [21]), which predicts cavities on the protein surface and performs docking of the ligand to these cavities using the software AutoDock Vina [25]. The software automatically prepares the ligand and target, removing non‐protein atoms for docking, and binding modes are then ranked according to the Vina score, and a 3D visualization of the binding mode is provided. The AKB‐9778 chemical structures file was from Pubchem (Compound CID: 46700782). For docking to PTP catalytic domains, AlphaFold predicted structures of VE‐PTP (AF‐P23467‐F1‐v4) and CD148 (AF‐Q12913‐F1‐v4) were used, together with X‐ray crystallographic structures of PTPRO (PDB ID: 2G59) and PTP1B (PDB ID: 4I8N). AlphaFold predicted structures were used in preference to some experimental structures (2CFV and 2NZ6) which have unmodeled regions. In all structures, the WPD loop adopts the closed conformation typical of an inhibitor‐bound conformation. High scoring protein‐ligand complexes were visualized in PyMOL 3.0 (Schrödinger), and 2D ligand‐protein interaction maps were generated in Discovery Studio (BIOVIA).

### 2.9 Protein Purification and Site‐Directed Mutagenesis

PTP catalytic domains were expressed and purified as described previously [24]. Site‐directed mutagenesis was performed using the QuikChange Site‐Directed Mutagenesis protocol (Agilent). Mutations (Tyr1071Ala, Gln1283Ala, His1206Ala, and Asn1073Ala) were introduced into the CD148 catalytic domain expression plasmid using complementary mutagenic primers (Supporting Information Data) containing the desired nucleotide substitutions.

### 2.10 Statistics

Data are presented as either mean ± SEM or ± SD as in the figure legend. Statistical tests were performed with GraphPad Prism 10.

### 2.11 Nomenclature of Targets and Ligands

Key protein targets and ligands in this article are hyperlinked to corresponding entries in http://www.guidetopharmacology.org, the common portal for data from the IUPHAR/BPS Guide to PHARMACOLOGY [26], and are permanently archived in the Concise Guide to PHARMACOLOGY 2025/26 [27].

## 3 Results

### 3.1 AKB‐9778 Partially Inhibits Platelet‐Dependent Thrombus Formation In Vitro

**FIGURE 1:**
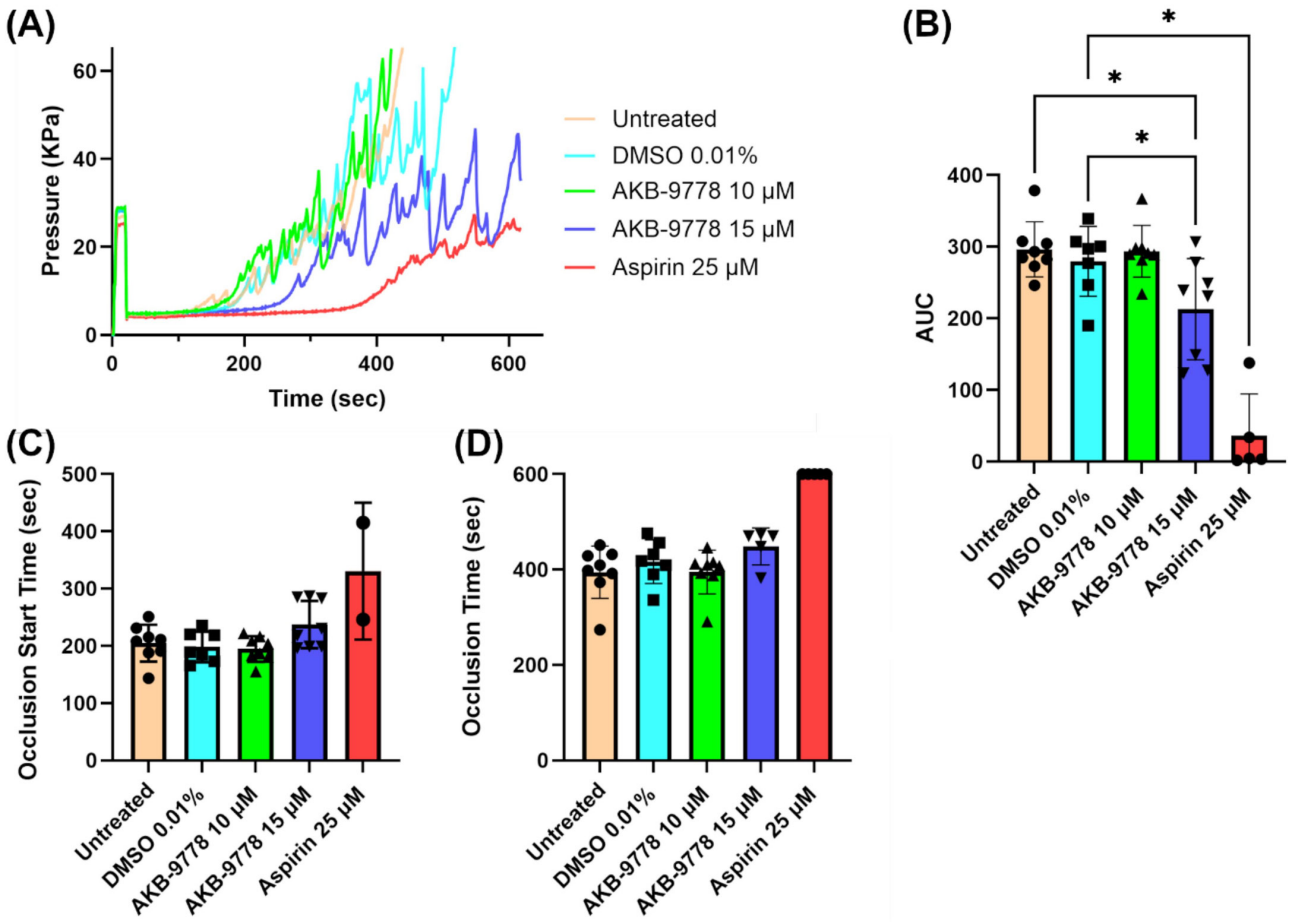
AKB‐9778 partially inhibits platelet‐dependent thrombus formation in vitro. (A) Representative flow pressure curves (< 60 kPa) for 10 min using the PL chip with a whole blood sample pre‐treated as indicated. (B) Mean area under the curve (AUC) values ± SD for the 10‐min period are shown for each treatment. (C) Mean occlusion start time ± SD. (D) Mean occlusion time ± SD. An occlusion start time was not recorded in 3 aspirin‐treated samples, and the occlusion time in all aspirin‐treated blood samples exceeded 600 s. Statistical analysis was performed using one‐way ANOVA followed by Tukey's multiple comparisons test in GraphPad Prism. *p* < 0.05 was considered statistically significant.

AKB‐9778 partially inhibited thrombus formation in whole blood as measured using the T‐TAS. Whole blood from healthy donors was analyzed using collagen‐coated PL chips at a shear rate of 1500 s^−1^, and thrombus formation was monitored by flow pressure changes (Figure 1). In control samples, occlusion start time occurred within 2–3 min and occlusion time ranged from 5 to 8 min. AKB‐9778 produced a concentration‐dependent reduction in the AUC (area under the flow pressure curve) which relates to overall thrombus formation. While 10 μM had no significant effect, treatment with 15 μM AKB‐9778 reduced the mean AUC10 by 28% (296 ± 39 vs. 213 ± 71) (Figure 1B). By comparison, aspirin (25 μM) reduced AUC10 by approximately 80%. No significant changes in occlusion start time or occlusion time were observed at the inhibitor concentrations tested (Figure 1C,D). In contrast, no significant effects were observed in AR chips containing collagen and thromboplastin (Supporting Information Data).

### 3.2 AKB‐9778 Does Not Alter Platelet Activation Markers in Response to Agonists

**FIGURE 2:**
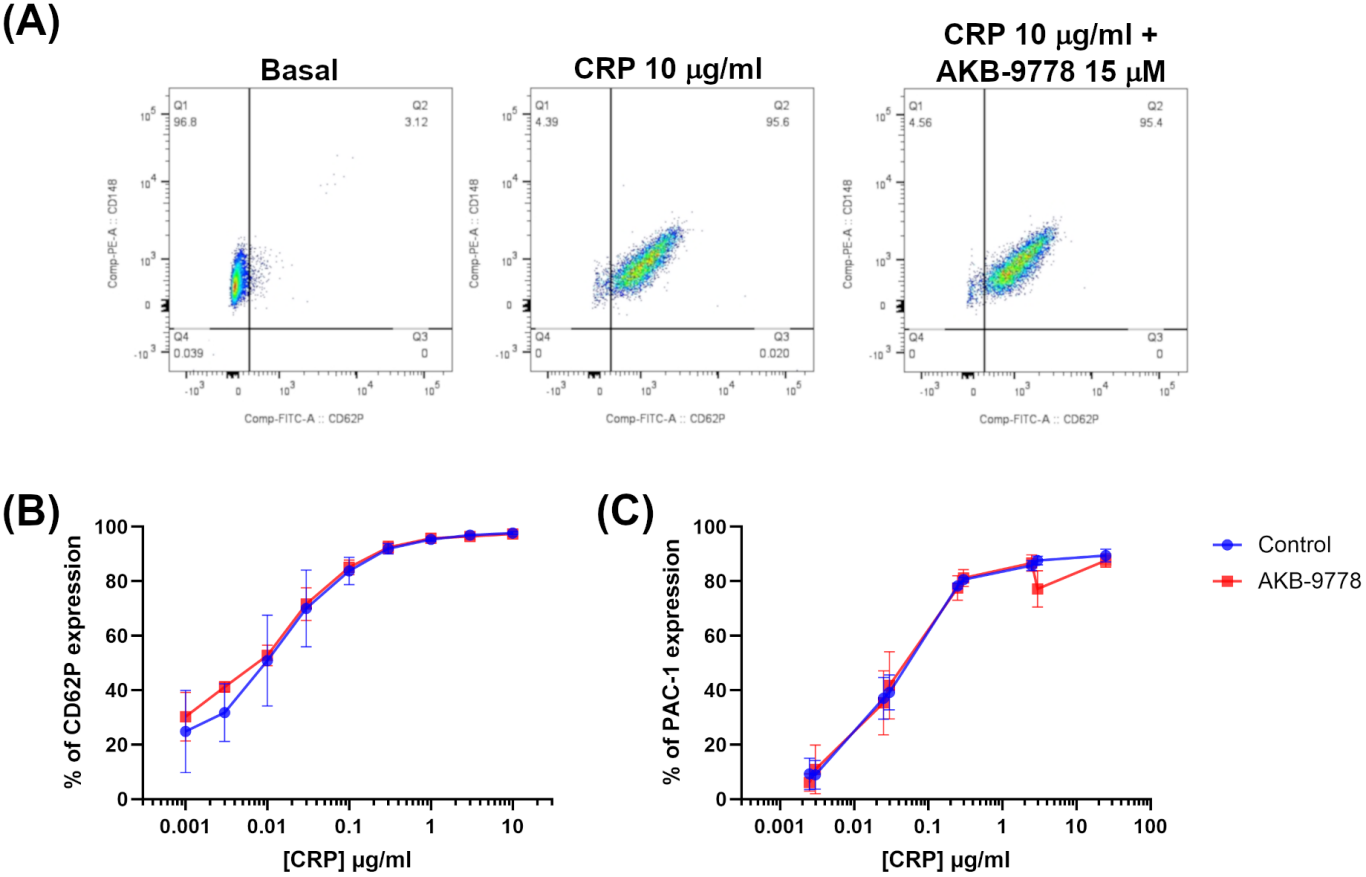
AKB‐9778 does not inhibit CRP‐stimulated platelet activation markers. (A) Comparative flow cytometry quadrant dot plots of CD148‐PE and CD62P‐FITC double‐stained platelets after either no treatment or stimulation with CRP (10 μg/mL) or CRP (10 μg/mL) in the presence of AKB‐9778 (15 μM). (B) CRP dose‐dependent stimulation of CD62P expression in the presence or absence of AKB‐9778 (15 μM). (C) CRP dose‐dependent stimulation of PAC‐1 expression in the presence or absence of AKB‐9778 (15 μM). Data are mean ± SEM, *n* = 3 experiments.

Flow cytometry analysis revealed that 15 μM AKB‐9778 did not affect CRP‐ or PAR1 peptide‐stimulated CD62P expression or binding of the PAC‐1 antibody to the active, high affinity conformation of the integrin αIIbβ3 (Figure 2), indicating that inhibition of thrombus formation is not mediated via direct suppression of these early platelet activation markers. CRP stimulated a dose‐dependent increase in CD62P expression and PAC‐1 antibody binding, both of which were unaffected by AKB‐9778 (15 μM) (Figure 2). Similarly, no significant effect of the inhibitor was observed when the PAR1 activating peptide was used (data not shown).

### 3.3 Predicted AKB‐9778 Binding Involves Key Residues in the PTP Catalytic Pocket

**FIGURE 3:**
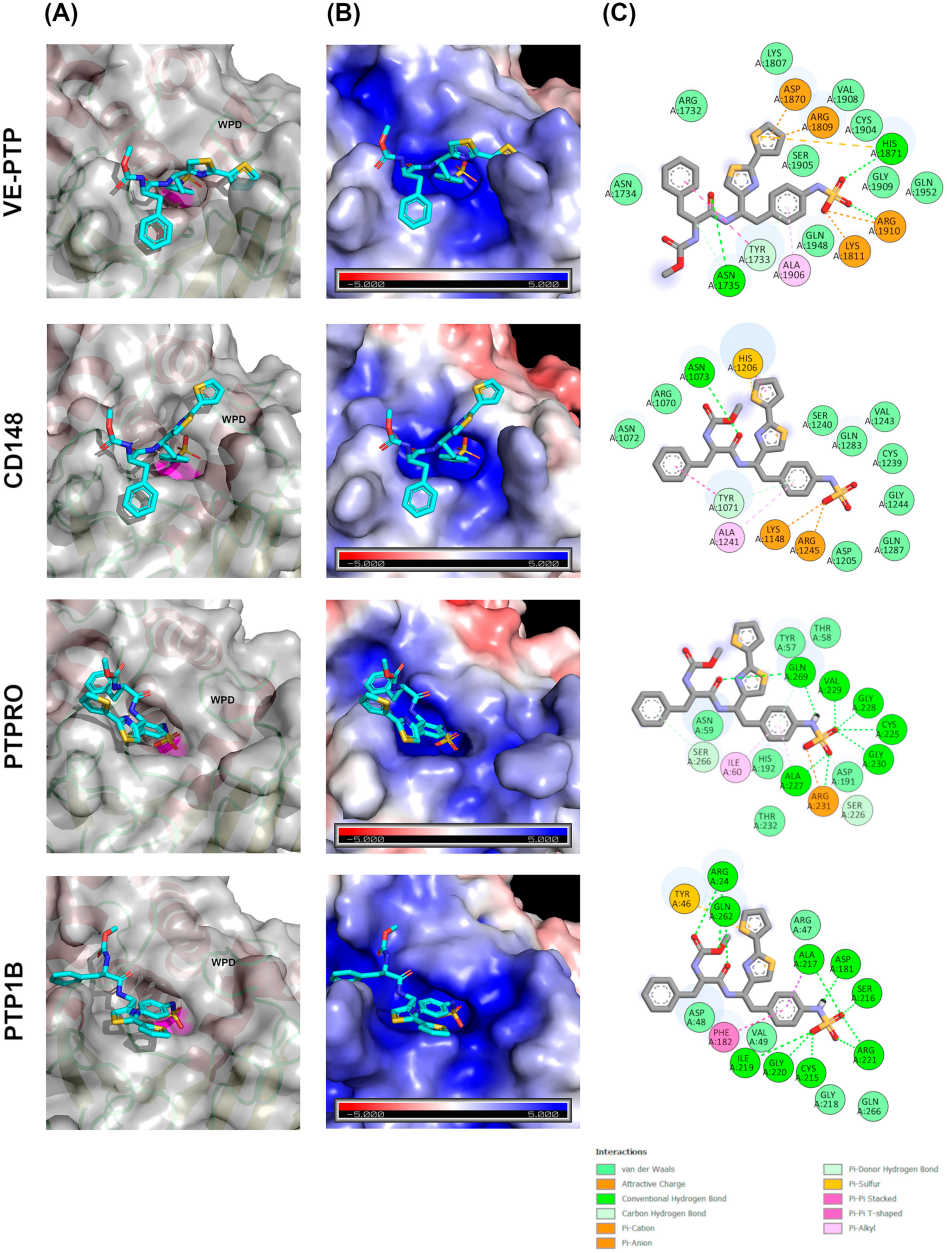
Molecular docking of AKB‐9778 to PTP catalytic domains. (A) A light gray semi‐transparent surface representation is shown for the region around the active site of each phosphatase with the catalytic cysteine colored magenta. The position of the WPD (Trp‐Pro‐Asp) loop (green) is shown. Inhibitor AKB‐9778 is colored (teal). (B) Protein surface electrostatic charge is shown calculated using PyMOL software with the Adaptive Poisson‐Boltzmann Solver plug‐in. Positive and negative charges are blue and red, respectively. (C) 2D ligand protein interaction diagrams are shown. Dashed lines represent interaction of ligand with amino acids and their color indicates the interaction type, as shown in the key. The blue halo surrounding some residues represents the solvent accessible surface.

Since the molecular basis of interactions between AKB‐9778 and VE‐PTP, or other protein tyrosine phosphatases, is poorly defined, we used molecular docking with CB‐Dock to investigate this in more detail. The CB‐Dock tool integrates curvature‐based cavity detection with docking via AutoDock Vina and ranks the binding modes according to the free energy of binding Vina score in kcal/mol. When running the CB‐Dock to search more than 10 cavities, several high‐scoring docking poses were returned when performing docking to VE‐PTP and other PTPs; however, since AKB‐9778 is a competitive inhibitor, we focused attention on those in which the inhibitor was bound in the active site (Figure 3). Docking analysis predicted that AKB‐9778 binds within the catalytic pocket of VE‐PTP with a Vina score of −7.2. The phenylsulfamic acid group of the inhibitor is orientated towards the active site cysteine (Cys1904), making hydrogen bonds and ionic interactions with Lys1811, Arg1910, and His1871. The thiophene ring forms π‐cation and π‐anion interactions with residues in the WPD loop, while the phenyl ring interacts with the phosphotyrosine recognition motif.

Molecular docking results are also shown for interaction of AKB‐9778 with CD148 (Vina score −6.5), PTPRO (Vina score −6.9), and PTP1B (Vina score −8.5), also with the phenylsulfamic acid group in the active site (Figure 3). Ligand binding is shown with a surface representation of each phosphatase alongside 2D interaction maps of ligand protein interactions.

**FIGURE 4:**
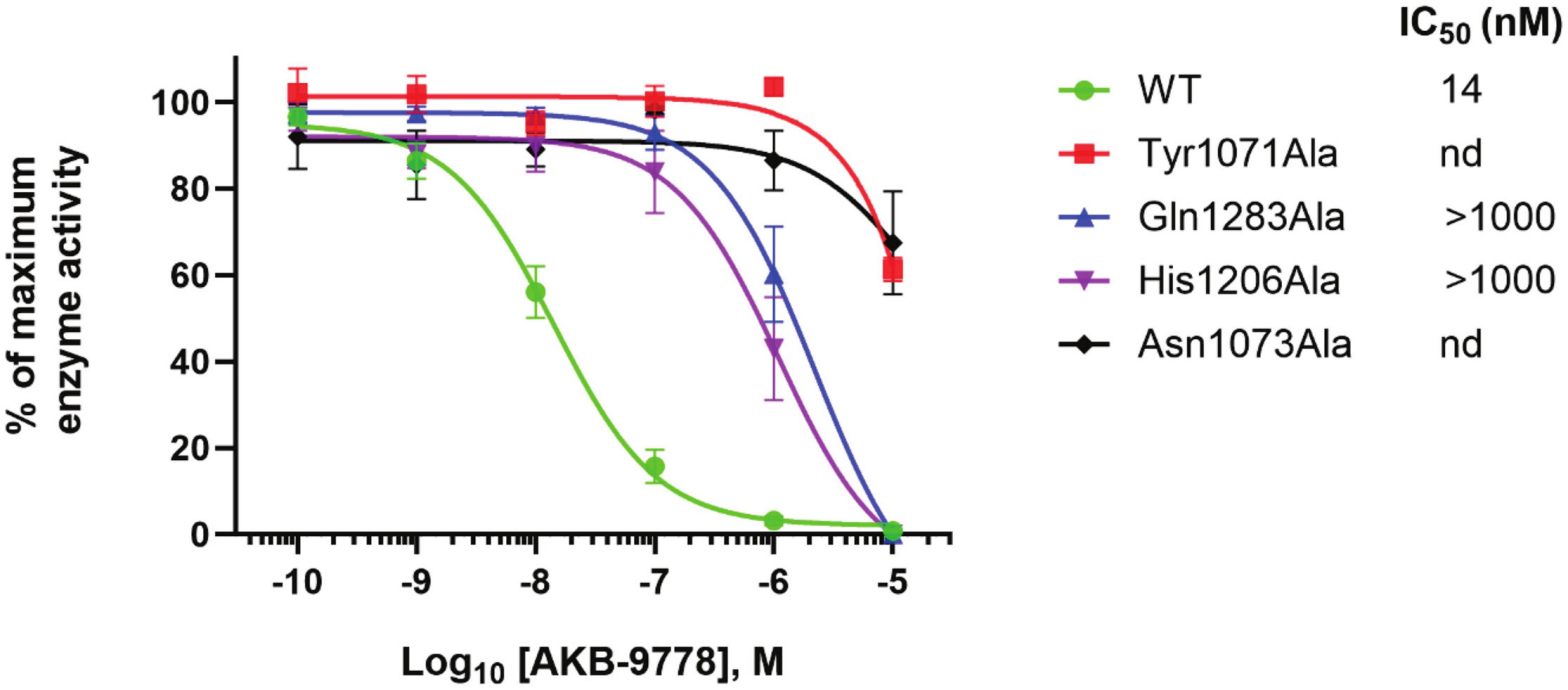
Reduction of AKB‐9778 inhibitory effect on CD148 by mutation of active site residues. Percentage inhibition of phosphatase activity relative to control from 3 separate experiments, mean ± SEM. IC_50_ values were determined by nonlinear regression in GraphPad Prism.

As CD148 is the focus of the present study, we used site‐directed mutagenesis to validate the inhibitor's predicted interactions. The predicted binding mode of the inhibitor involves aromatic *π*—*π* offset stacking interactions between the thiophene ring of AKB‐9778 and the imidazole ring of His1206 (3.8 Å) with π‐sulfur interactions (3.7 Å) and T‐shaped *π*—*π* interaction between the benzene ring and Tyr1071 (4.8 Å). The sulfamic acid group is predicted to make a hydrogen bond (3.4 Å) with the sidechain of Gln1283 and a network of other charge–charge interactions with the bottom of the positively charged active site pocket. While the sidechain nitrogen of Asn1073 forms hydrogen bonds with the oxygen atoms of the carbonyl groups in the inhibitor (Figure 3). Mutation of each residue resulted in a > 100‐fold increase in IC_50_, indicating that these residues contribute to inhibitor binding (Figure 4).

### 3.4 AKB‐9778 Selectively Inhibits R3 Subgroup Phosphatases In Vitro

**FIGURE 5:**
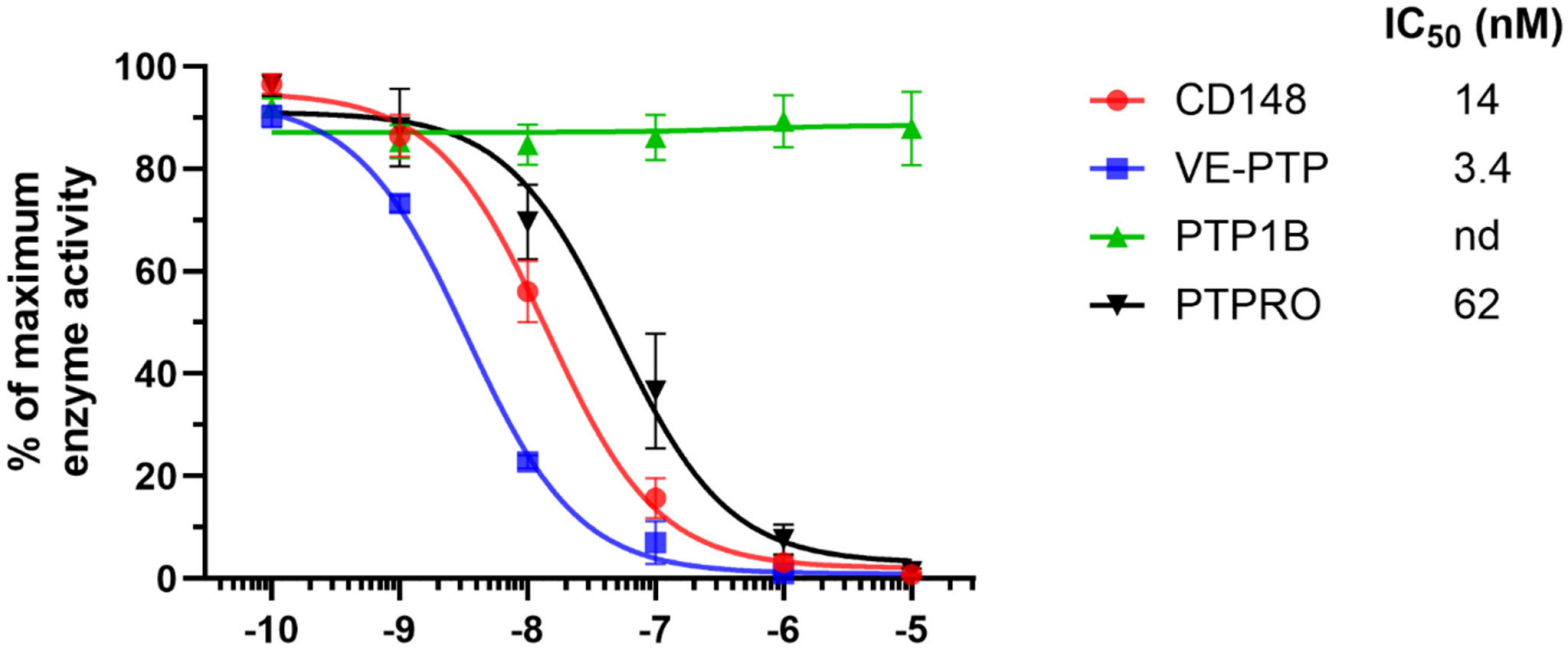
AKB‐9778 inhibition of R3 subgroup PTP activity in vitro. Percentage inhibition of phosphatase activity relative to control from 3 separate experiments, mean ± SD. Curve fitting was performed with GraphPad Prism.

To verify the inhibitory activity of AKB‐9778 and determine whether the predicted interactions translated into functional inhibition, we assessed the activity of AKB‐9778 against several PTP catalytic domains using the artificial fluorescent substrate DiFMUP. Initial experiments determined suitable enzyme concentrations to achieve a linear reaction rate over 30 min and a substrate concentration corresponding to the *K*_*m*_ was used (Supporting Information Data). AKB‐9778 inhibited VE‐PTP with high potency (IC_50_ = 3.4 nM). In contrast, inhibition of CD148 and PTPRO was weaker, with IC_50_ values of 14 nM and 62 nM, respectively (Figure 5). No significant inhibition of PTP1B was observed at concentrations up to 10 μM.

## 4 Discussion

Our study demonstrates that AKB‐9778 (razuprotafib) partially inhibits platelet‐dependent thrombus formation on collagen in vitro, likely through inhibition of the receptor‐type PTP CD148. In collagen‐coated PL chip assays, 15 μM AKB‐9778 reduced the area under the flow pressure curve (AUC10) by 28%, while 10 μM had no significant effect, consistent with concentration‐dependent activity. In contrast, no significant effect was observed in AR chips containing collagen and thromboplastin, indicating that AKB‐9778 does not affect coagulation and that its action is primarily at the level of platelet function. To investigate the mechanistic basis of the observed effects we used flow cytometry to study platelet activation, molecular docking, site‐directed mutagenesis and phosphatase assays to gain insight into the inhibitory activity.

Flow cytometry analysis revealed that AKB‐9778 did not alter CRP‐ or PAR1‐stimulated CD62P expression or PAC‐1 binding, which are markers of early platelet activation. We tested the inhibitor over a range of agonist concentrations because studies of CD148 KO mice have demonstrated that the effect of loss of CD148 function can be overcome by high concentrations of agonist [1]. The lack of effect likely reflects the partial inhibition of CD148 by the inhibitor. These findings are consistent with studies in CD148 knockout mice, where complete loss of CD148 significantly impairs platelet activation, whereas partial expression restores platelet function.

Molecular docking provided insight into the predicted binding of AKB‐9778 to PTP catalytic domains, in the absence of any reported co‐crystal structures. For CD148, several high‐scoring poses of protein‐ligand complexes were obtained in which the phenylsulfamic acid group of the inhibitor was oriented towards the catalytic cysteine. These multiple configurations likely reflect the flexibility of the inhibitor, which has 12 rotatable bonds. The functional importance of interactions involving Tyr1071, His1206, Gln1283, and Asn1073 in CD148 was confirmed by site‐directed mutagenesis of these residues, with each mutation increasing the IC_50_ of AKB‐9778 by > 100‐fold. These results support a competitive inhibitory mechanism and provide a structural explanation for the partial inhibition observed in platelets.

In vitro phosphatase assays further characterized the selectivity of AKB‐9778. The inhibitor potently suppressed VE‐PTP activity (IC_50_ = 3.4 nM) but was less effective against CD148 (IC_50_ = 14 nM) and PTPRO (IC_50_ = 62 nM), while PTP1B remained unaffected at concentrations up to 10 μM. Notably, these IC_50_ values are substantially higher than originally reported (VE‐PTP: 0.017 nM; CD148: 0.036 nM), likely reflecting differences in assay conditions or compound batches, and suggesting that the 15 μM concentration used in cell‐based assays may not fully inhibit CD148. We tested the inhibitor from two different chemical reagent suppliers but were unable to replicate the originally reported IC_50_ values. The general PTP inhibitor sodium orthovanadate inhibited as expected with an IC_50_ for PTP1B corresponding to the published value (Supporting Information Data). Other studies [18] have also reported a wide variation in IC_50_ values from those originally reported.

Our results reinforce the potential of CD148 as an anti‐thrombotic target. Thrombotic cardiovascular diseases are a leading cause of deaths worldwide [27]; however, current anti‐thrombotic drugs, including anti‐platelet drugs and anti‐coagulants, are associated with a significant bleeding risk which increases morbidity and mortality. Evidence suggests that the molecular and cellular mechanisms of hemostasis and thrombosis can be separated, thereby enabling development of what has been referred to as the ‘holy grail’, new antithrombotic therapeutics with reduced bleeding risk [28, 29]. Anti‐thrombotic targets that may spare hemostasis have been suggested in the coagulation system, leukocyte‐platelet interface and in platelets [28], including CD148 [1, 2, 3, 4]. Strategies that selectively inhibit platelet‐mediated thrombus formation while sparing hemostasis are highly desirable. CD148 inhibition may represent such a strategy, as supported by evidence from knockout mice and human studies, where loss of CD148 function impairs thrombus formation without affecting normal hemostasis.

A limitation of the current study is the modest effect of AKB‐9778 on thrombus formation, likely due to partial inhibition of CD148 and limited cell permeability. Also, the inhibitor lacks selectivity against other R3 subgroup PTPs; however, it should be noted that platelets do not express VE‐PTP and have low levels of PTPRO. PTPRO‐deficient mice have no platelet phenotype (Y. Senis unpublished); therefore, an inhibitory mechanism involving these phosphatases can be ruled out. CD148 is known to be essential for tyrosine‐kinase‐linked receptor‐mediated aggregation; however, this has not been investigated in the present study. Since CD148 is a phosphatase, further work analyzing phosphorylation kinetics of key targets such as LAT and PLCγ could provide further mechanistic insights. Also, since loss‐of‐function SNPs in *PTPRJ* reduce FcγRIIA‐dependent platelet aggregation, it would be informative to determine if a similar effect is observed with AKB‐9778. Given the wide variation in IC_50_ values between the original report and other work, direct measurement of affinity of the inhibitor using biophysical assays would be valuable. Future work should focus on developing more potent, platelet‐targeted CD148 inhibitors with improved pharmacological properties. Such agents have the potential to achieve clinically relevant anti‐thrombotic effects while minimizing bleeding risk.

In conclusion, AKB‐9778 partially inhibits in vitro thrombus formation via CD148, and our structural and functional analyses provide a foundation for the design of more selective and efficacious CD148‐targeted anti‐thrombotic therapies.

## Author Contributions

A.J.B. and Y.A.S. conceived of the project. L.E.B. and A.J.B. wrote the manuscript. L.E.B. carried out the experiments and analyzed the data with assistance from S.J. All authors discussed the results and contributed to the final manuscript.

## Funding

This study was supported by UKRI QR funding to the University of Westminster and the Professor Geoffrey Petts Memorial Fund.

## Ethics Statement

Protocols were approved by the Ethical Review Board of the University of Westminster (ETH2122‐0605).

## Consent

The blood donors provided written informed consent.

## Conflicts of Interest

The authors declare no conflicts of interest.

## Supporting information


**Data S1:** prp270301‐sup‐0001‐Supinfo.docx.

## Data Availability

The data that support the findings of this study are available from the corresponding author upon reasonable request.
