# Supplementary material for: Targeting CD148 for Antithrombotic Therapy: Functional and Molecular Evaluation of AKB‐9778
Source: Pharmacol Res Perspect. 2026 Jul 31;14(4):e70301. doi: 10.1002/prp2.70301 (PMC13428021; doi:10.1002/prp2.70301)
Supplement: Supplementary file 1 — Data S1: prp270301‐sup‐0001‐Supinfo.docx. [file PRP2-14-e70301-s001.docx]

**SUPPLEMENTARY DATA**

**1. T-TAS data with AKB-9778 using AR chips**

**
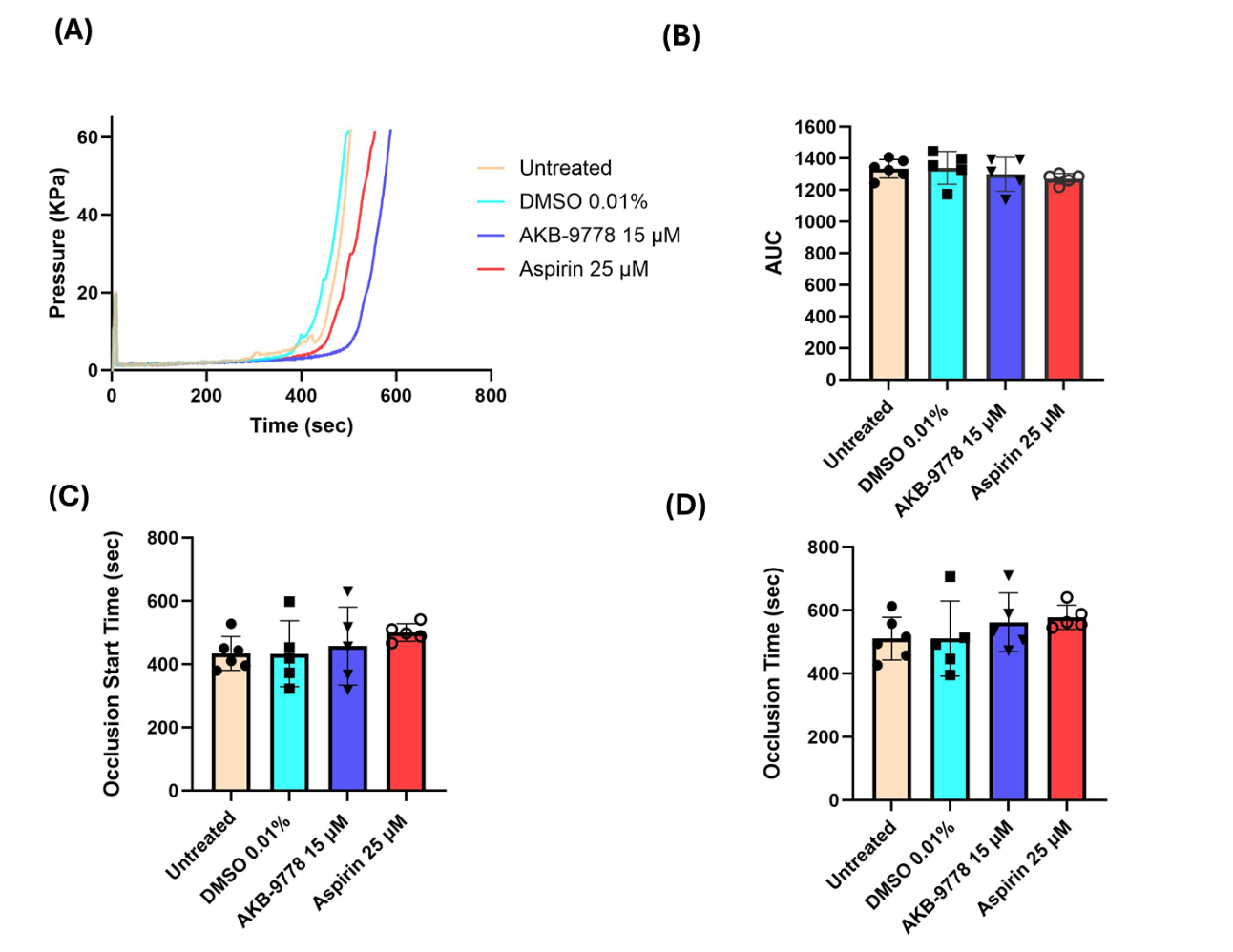
**

**2. Calculation of K_m_ for PTPs**

**
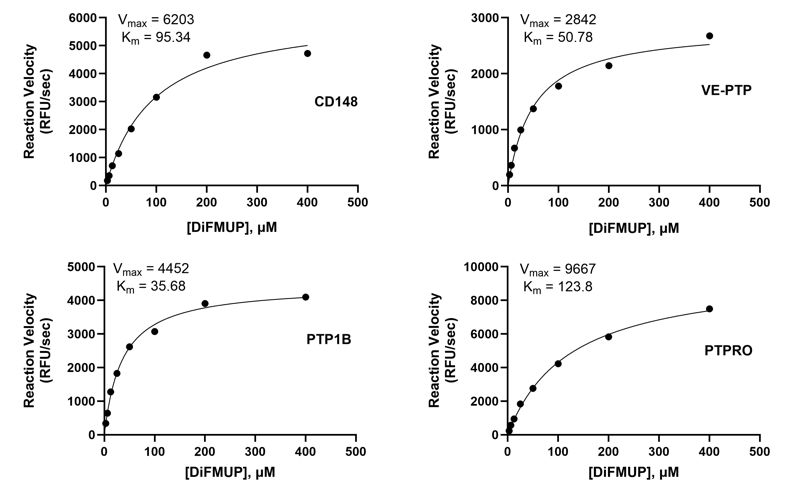
**

**3. Effect of orthovanadate on phosphatase activity**


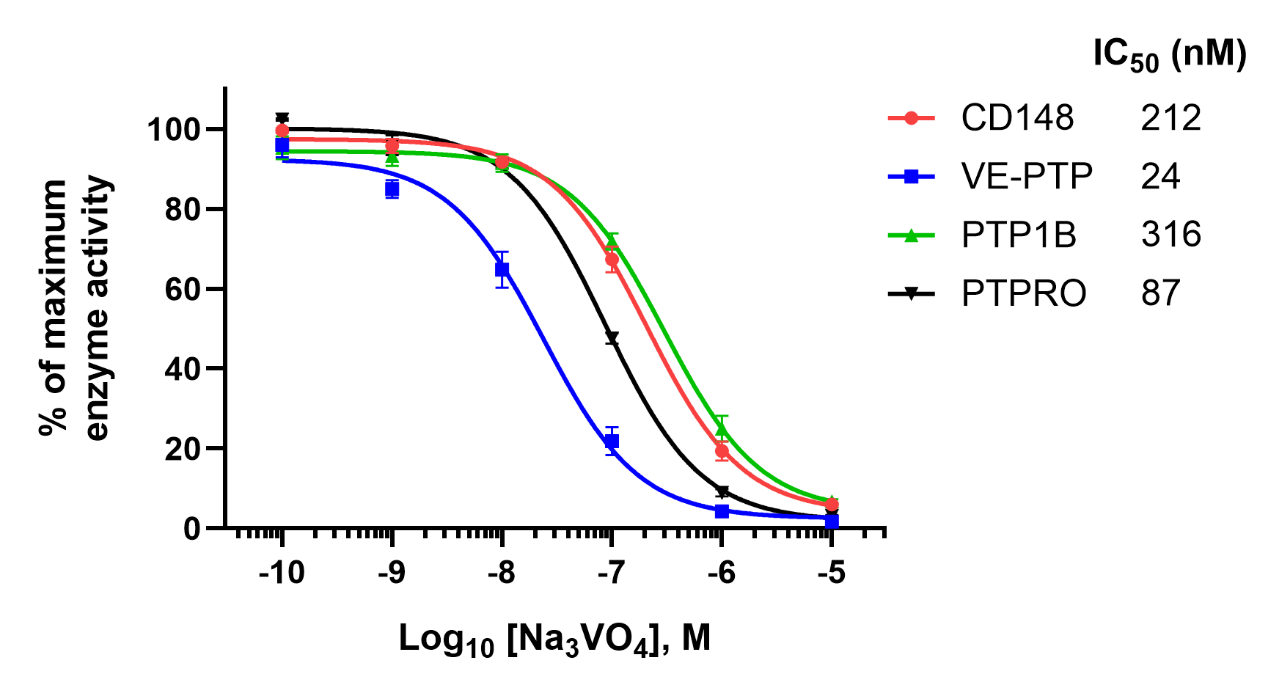


**4. Oligonucleotide primer pairs for site-directed mutagenesis**

| Tyr1071Ala | t267g_a268 c_F | ATATCATAGGGCAGAACATTATTAGCGCGATTCTTTCCTCTATTCTCAGC |
| --- | --- | --- |
|  | t267g_a268 c_R | GCTGAGAATAGAGGAAAGAATCGCGCTAATAATGTTCTGCCCTATGATAT |
| Gln1283Ala | c903g_a904 c_F | AACATACTGGTCCTCTGTCGCCACCATTAAAGGCCTATGC |
|  | c903g_a904 c_R | GCATAGGCCTTTAATGGTGGCGACAGAGGACCAGTATGTT |
| His1206Ala | c672g_a673 c_F | GTCGGGAACACCGGCGTCTGGCCAGGAG |
|  | c672g_a673 c_R | CTCCTGGCCAGACGCCGGTGTTCCCGAC |
| Asn1073Ala | a273g_a274 c_F | ATCATAGGGCAGAACAGCATTATAGCGATTCTTTCCTCTATTCTCAGCC |
|  | a273g_a274 c_R | GGCTGAGAATAGAGGAAAGAATCGCTATAATGCTGTTCTGCCCTATGAT |
